# Genome-Wide Identification and Analysis of Ariadne Gene Family Reveal Its Genetic Effects on Agronomic Traits of *Brassica napus*

**DOI:** 10.3390/ijms23116265

**Published:** 2022-06-03

**Authors:** Sumbal Wahid, Meili Xie, Sehrish Sarfraz, Jie Liu, Chuanji Zhao, Zetao Bai, Chaobo Tong, Xiaohui Cheng, Feng Gao, Shengyi Liu

**Affiliations:** Key Laboratory of Biology and Genetics Improvement of Oil Crops, Oil Crops Research Institute of Chinese Academy of Agricultural Sciences, Ministry of Agriculture and Rural Affairs, Wuhan 430062, China; sumbalwahid@gmail.com (S.W.); sehrishsarfraz04@gmail.com (S.S.); whjiejiel@163.com (J.L.); zhaochuanji@caas.cn (C.Z.); baizetao_2005@163.com (Z.B.); tongchaobo@126.com (C.T.); cxh5495@163.com (X.C.); liusy@oilcrops.cn (S.L.)

**Keywords:** E3 ligases, Ariadne (ARI) proteins, ubiquitination, *AtARI8*, phylogenetic analysis, association mapping analysis, agronomic traits, *B. napus*

## Abstract

E3 ligases promote protein ubiquitination and degradation, which regulate every aspect of eukaryotic life. The Ariadne (ARI) proteins of RBR (ring between ring fingers) protein subfamily has been discovered as a group of potential E3 ubiquitin ligases. Only a few available research studies show their role in plant adaptations processes against the external environment. Presently, the functions of ARI proteins are largely unknown in plants. Therefore, in this study, we performed genome-wide analysis to identify the *ARI* gene family and explore their potential importance in *B. napus*. A total of 39 *ARI* genes were identified in the *B. napus* genome and were classified into three subfamilies (A, B and C) based on phylogenetic analysis. The protein–protein interaction networks and enrichment analysis indicated that *BnARI* genes could be involved in endoreduplication, DNA repair, proteasome assembly, ubiquitination, protein kinase activity and stress adaptation. The transcriptome data analysis in various tissues provided us an indication of some *BnARI* genes’ functional importance in tissue development. We also identified potential *BnARI* genes that were significantly responsive towards the abiotic stresses. Furthermore, eight *BnARI* genes were identified as candidate genes for multiple agronomic traits through association mapping analysis in *B. napus*; among them, *BnaA02g12100D*, which is the ortholog of *AtARI8*, was significantly associated with ten agronomic traits. This study provided useful information on *BnARI* genes, which could aid targeted functional research and genetic improvement for breeding in *B. napus*.

## 1. Introduction

Ubiquitination is one of the important mechanisms that prepare the cell response toward internal and external stimuli during plant life [1]. It is the process of protein degradation in which the ubiquitin-26S proteasome system (UPS) is the main player [2]. The targeted proteins are first bound to the multiple ubiquitin (Ub) proteins. Ub is a 76-aminoacid protein that is a highly conserved housekeeping protein in all the eukaryotes. A series of enzymes, including ubiquitin-protein ligases (E3), ubiquitin-activating enzymes (E1) and ubiquitin-conjugating enzymes (E2 or Ubc), are used to transfer Ub to a target protein. Firstly, the Ub protein is activated by interacting with E1 and forms a E1-Ub molecule at the expense of ATP molecules. This activated Ub molecule is transferred to E2 through the interaction of E1-Ub with E2 and forms an intermediate complex, E2-Ub. Then, the E3 covalently attaches the Ub with the target protein by interacting with the E2-Ub intermediate complex and target protein. In this way, many targeted proteins are bound to Ub proteins binds by the repetition of the above-mentioned process, and hence are recognized and degraded by the 26S proteasome [1,2,3]. About 6% of *A. thaliana* proteome constitutes this proteasome system [4]. Among E1, E2 and E3, E3 are most abundant in eukaryotes and 1400 E3s are predicted in *A. thaliana* [5]. By far, E3s are of more importance because they are diverse, directly interacting with the conjugated Ub and binding to the target protein, therefore maintaining specificity [6]. These E3 ligases maintain the cellular response by targeting cell division, signaling, immune responses and DNA repair [7].

In general, E3 ligases are divided into two groups, each of which has either a HECT domain or a RING (really interesting new gene) domain [7,8]. The RING domain containing E3s is usually identified with a cysteine-histidine rich ring motif with Zinc atoms [7]. It is one of the most detected domains in *A. thaliana* [9] and it has been involved in protein–protein interaction processes [10]. In Arabidopsis and rice genomes, 426 and 425 RING type E3 ligases have been identified, respectively [6,11]. The RING E3 ligases were responsive towards dehydration, cold and heat treatment in rice leaf tissue [12]. AIRP2 (ABA insensitive RING protein 2) is also a E3 ring type ligase which was involved in Abscisic-Acid-mediated drought tolerance in Arabidopsis [13]. RING E3 ligases also play an important role in the plasticity of the flowering time of plants; for example, the photoperiodic pathways are regulated through ubiquitination and, hence, timely floral induction is ensured [14].

One of the largest group of proteins that contain the RING domain is RBR (ring between ring fingers) proteins, identified by the presence of their RING1-IBR-RING2 domains (RBR supra domain) [15]. It constitutes RING1 at the N-terminal, an IBR (in between ring), and RING2 at the C-terminal. E3 ligase activity is seen in several RBR proteins [15]. The plant RBR proteins consist of Ariadne (ARI proteins), ARA54, Plant II and Helicase subfamilies [16]. The best known RBR protein is Parkin, which is involved in autosomal recessive familial Parkinson’s disease [17,18], and the Ariadne family shares its RBR domain similarity [17,19]. The RING1 is a typical RING finger with a C3HC4 signature of conserved cysteine and histidine residues, the IBR domain has a typical C6HC signature, while the RING2 is shorter than the canonical RING structure. In addition to the RBR domain, ARI proteins also contain the Ariadne domain at the C-terminus [17,20].

The *ARI* gene family has been identified in human [21,22,23], Drosophila [24], mouse [25], and Arabidopsis [9]. However, the mechanism and functions of ARI proteins are largely unknown, for only limited literature is available regarding the functional importance of these genes. Some ARI proteins are involved in E2 enzyme interactions in Drosophila [24] and human [23]. Ubiquitination activity is only proven in *ARI8* in *A. thaliana* [6]. *AtARI12* was involved in UV- B pathway through interaction with Constitutively Photomorphogenic 1 (COP1), which is a key component of the light signaling pathway [26,27]. The overexpression of a soybean Ariadne-like gene caused aluminum tolerance in Arabidopsis [28]. The orthologous gene of *AtARI7* in *Hypericum perforatum* was associated with apospory [29].

*Brassica napus*, an allotetraploid plant generated through hybridization between *Brassica rapa* and *Brassica oleracea* [30], is a major worldwide crop due its industrial value and oil production. Like other crops, *B. napus* productivity has been affected by several abiotic stresses including low temperature, drought, salinity, and so on [31,32,33]. Therefore, it is important to improve stress adaptation by identifying stress-related genes in *B. napus*. To date, genome-resource and various transcriptome datasets have been published [34,35]. However, *ARI* gene family members and their potential importance have not been investigated in *B. napus*. Therefore, a genome-wide study was designed to find out the number of genes in this family. The evolutionary relationships, gene architectures, conserved motifs, gene duplications, and protein–protein interactions in *B. napus* were all investigated in this work. Diverse tissues and environmental stressors transcriptome data was used to understand the expression patterns of *ARI* genes in *B. napus.* Furthermore, we also investigated the genetic variations (SNPs) in the *BnARI* gene family and associated them with several agronomic traits in the natural population of *B. napus*. This research enriched our knowledge of *BnARI* genes and laid the groundwork for future functional research and genetic breeding of *B. napus*.

## 2. Results

### 2.1. Identification of ARI Genes in B. napus

We identified 39 *ARI* genes in the *B. napus* genome by querying 16 *A. thaliana* ARI protein sequences (Table 1 and Table S1). All the BnARI proteins contained the RBR and Ariadne domains. The detailed information of *BnARI* genes is summarized in Table 1. The protein length varied from 369 to 672 amino acids and the molecular weight ranged from 40.5 to 75.1 kDa. The isoelectric points increased from 4.7 to 6.48 (Table 1). Based on CELLO server prediction, 37 ARI proteins were localized in the nucleus (Table 1).

### 2.2. Phylogenetic Analysis of BnARI Proteins

**Figure 1:**
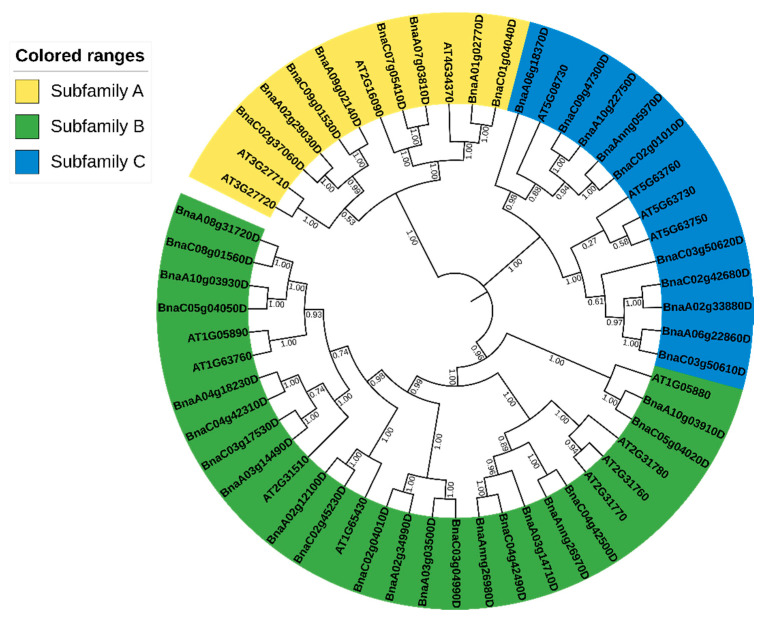
Phylogenetic analysis of ARI proteins in *B. napus* and *A. thaliana*. All ARI proteins are grouped into three subfamilies, and each subfamily is represented by a different color. The number on the branches shows the bootstrap values.

To determine the evolutionary relationship between *BnARI* and *AtARI* genes, a phylogenetic tree based on the NJ (neighbor-joining) method with 1000 bootstrap replications was constructed using their protein sequences. A total of 16 *AtARIs* and 39 *BnARIs* were clustered into three subfamilies (Figure 1, Table 1). In addition, in each subfamily, the *BnARIs* were clustered with their closest homologous gene in *A. thaliana*. Based on the previous nomenclature system used in *A. thaliana*, these subfamilies were named subfamily A, B and C, respectively (Figure 1, Table 1). Subfamily B was the largest and included 21 *ARI* genes, while Subfamily C and A contained 10 and 8 *ARI* genes, respectively.

### 2.3. Chromosomal Location and ARI Genes Duplication in B. napus

**Figure 2:**
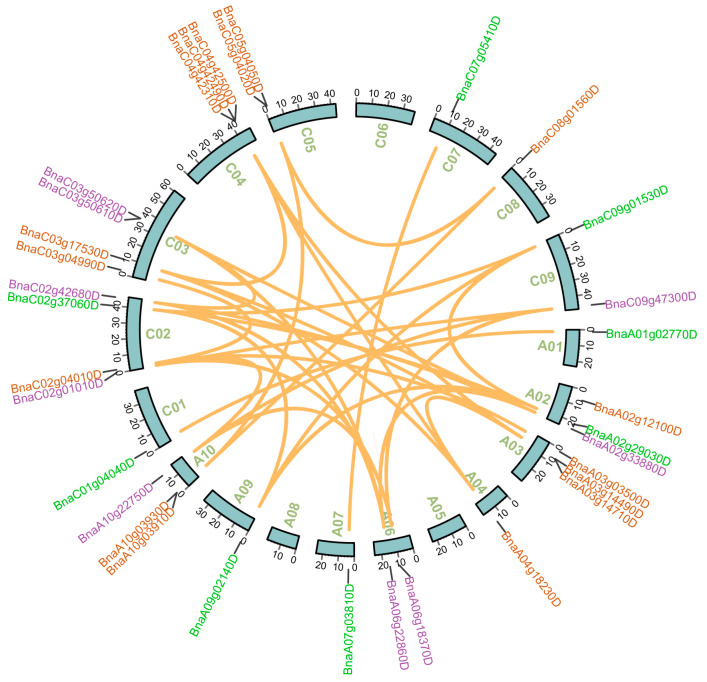
The chromosomal locations and duplicated genes analysis of *ARI* genes in *B. napus*. The locations of all the chromosomal *BnARI* genes are represented on different chromosomes, excluding the random fragment chromosomes. The different colors mean different *BnARI* subfamilies genes. Subfamily A, subfamily B and subfamily C are indicated by green, brown and purple color, respectively. The orange lines are used to highlight the duplicated *BnARI* gene pairs.

In *B. napus*, 33 *ARI* genes were unevenly distributed on the 19 chromosomes; the remaining six genes were located on random chromosomes (Table 1, Figure 2). A total of 20 and 19 *ARIs* were located on the A and C subgenomes, respectively. Chromosome C03 had the most *ARIs* (4 genes) while chromosomes A01, A04, A07, A09, C01, C07 and C08 had a single *ARI* gene. Chromosomes A05, A08, and C06 did not contain any *ARI* genes.

The expansion of the gene family is contributed by duplication events in the plant genome [36]. Therefore, the duplication events were analyzed for *ARI* genes. Based on BLAST and MCScanX analysis, the results showed that 35 out of 39 *ARIs* resulted from whole-genome duplication (WGD) or segmental duplication, while four genes were derived from tandem duplication (Table 1). Furthermore, 17 duplicated *ARI* pairs between subgenomes were found in *B. napus* (Figure 2; Table S2). To determine the selection pressure on these duplicated pairs during the evolution, the ratios of non-synonymous to synonymous substitutions (Ka/Ks) and divergence time were determined. The Ka/Ks value ranged from 0.038 to 0.035 with an average of 0.268 between gene pairs. The Ka/Ks ratio was significantly less than 1. The estimated divergence time of the duplicated pairs ranged from 1.509 Mya to 10.101 MYA with an average of 3. 877 MYA time period. The results indicated that *BnARIs* were under strong purifying selection (Table S2).

### 2.4. Gene Structure and Conserved Motif Analysis of BnARIs

**Figure 3:**
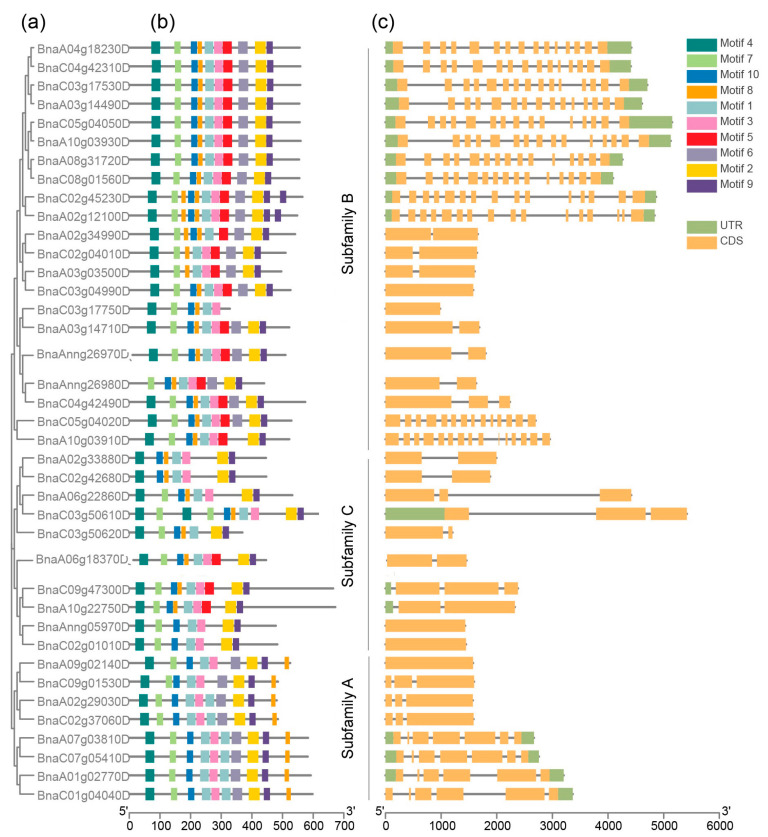
The phylogenetic relationship, conserved motifs and gene structure of *ARI* genes in *B. napus*. (**a**) The phylogenetic relationship of BnARI proteins. (**b**) The motif composition of BnARI proteins. Motifs (1–10) are shown in different colored boxes. (**c**) Gene structures of the BnARI genes. Orange boxes represent the CDS whereas green boxes represent UTR.

The exons and introns were examined to obtain insights into structural evolution in the *BnARI* gene family. The gene structure information was retrieved from the “*Darmor*-bzh” genome file in the BnaOmics database (Available online: https://bnaomics.ocri-genomics.net, accessed on 6 February 2022) using TBtool software. On average, each gene contained six exons but the number of exons of *BnARIs* greatly varied and ranged from 1 to 15 (Table 1, Figure 3). All the three subfamilies possessed a different number of exons; for example, subfamily C had the lowest number of exons (one–four), subfamily A contained up to seven exons while the subfamily B exhibited diverse exon number variations ranging from 1 to 15 (Table 1, Figure 3).

Furthermore, we extracted the protein data of BnARIs from the Protein FASTA file of *B. napus* in the BnaOmics database (Available online: https://bnaomics.ocri-genomics.net, accessed on 6 February 2022) to investigate the motif composition of all the *BnARIs.* The online MEME sever [37] was used to perform the motif analysis. Motifs 1, 5 and 8 were annotated as IBR; 2 and 6 were annotated as Ariadne domain; and motif 7 was annotated as Zinc finger (Table S3). Motifs 1, 2 and 7 were present in all the *BnARIs* except *BnaA02g33880D* and *BnaC02g42680D* which do not contain motif 7 (Figure 3). Both Ariadne domains (motifs 2 and 6) were present in subfamily A and B, whereas motif 6 was absent in subfamily C. Subfamily B and C both contained motif 1, 7, 8, 5 and 2 in principle but the only difference was subfamily C do not contain motif 6. The remaining motifs, 3, 4, 9, 10, were unknown domains but these were distributed evenly within the same subfamily (Figure 3). The motif arrangement in each subfamily also verified the phylogeny classification (Figure 1 and Figure 3).

### 2.5. Cis-Elements and Protein Interaction Analysis of BnARI Genes

**Figure 4:**
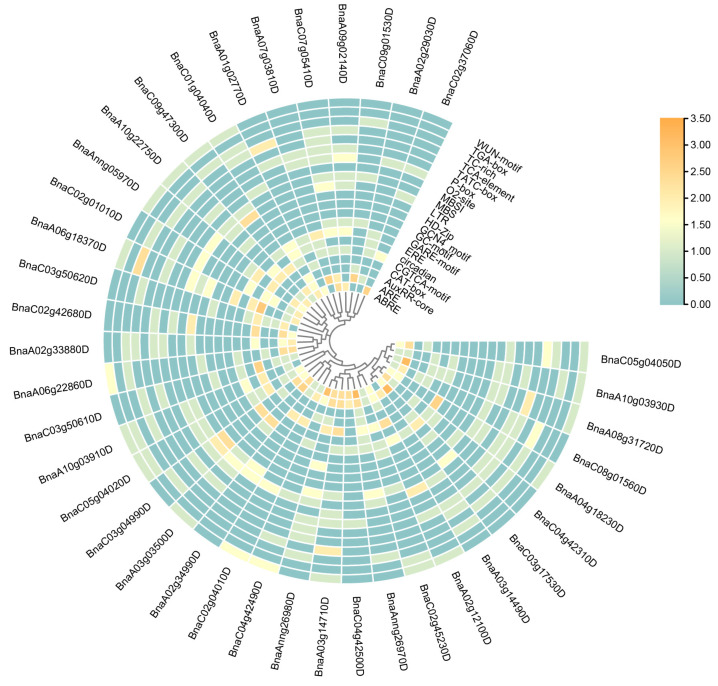
Cis-acting regulatory elements identified in *ARI* gene promoters in *B. napus*. The color bar shows log2 of copy number of cis-elements from low (cadetblue) to high (orange).

To determine the potential function of these *BnARIs*, we further analyzed the cis-elements in their promoter regions and proteins that could interact with them by using online public databases [38,39]. The 2kb upstream region of *BnARIs* were retrieved from the “*Darmor*-bzh” genome file and were analyzed for cis-elements through the PlantCARE database. These promoters were mainly enriched in growth-related and stress-related elements (Figure 4, Tables S4 and S5). ABRE (an element responsive to abscisic acid), CGTCA-motif (an element responsive to methyl jasmonic acid), ERE (an element response to ethylene), TCA-element (an element responsive to salicylic acid), P-box, GARE, AuxRR (an element responsive to auxin), TATC-box (an element responsive to gibberellin) and TGA-element (an element responsive to auxin) were hormone-related cis-regulatory elements and were listed according to their abundance (Figure 4, Table S4). Other growth-related elements included Circadian, O2-site (zein-metabolism-responsive element), CAT-box (a meristem-related element), MBSI (flavonoid-synthesizing element), HD-Zip (cell-differentiation element) and GCN4_motifs (an endosperm-development element); these elements are also presented according to their abundance (Figure 4, Table S4).

Stress-related elements were predominant in *ARIs* promoters and these included ARE (an element that responds to anaerobic induction), LTR (an element responsive to low temperature), MBS (the MYB binding site for drought stress), WUN-motif (an element responsive to wounds), TC-rich (a defense-responsive element), and GC-motif (an anoxic-specific element) (Figure 4, Table S4). Among all the promoter cis-elements, ARE was most abundant, whereas 33/39 of *BnARIs* contained ABREs (Figure 4, Table S4).

In subfamily A, a large number of stress- and hormone-related cis-acting elements were identified in the promoters of *BnARI* genes: ARE, ABRE and ERE elements had maximum copies and were detected in 5, 5 and 6 of the 8 *BnARI* gene promoters, respectively (Figure 4, Tables S4 and S5). The subfamily B members were enriched with stress-related element such as ARE, LTR, MBS and the WUN motif and at least 12 of 21 genes contain these elements. All 21 genes of this subfamily had the ARE motif, with an average of three copies. In 10 genes of subfamily C, CGTCA (on average, three copies), ARE (on average, two copies) and ABRE (on average, two copies) motif were found in 9, 10 and 9 genes, respectively. Stress-related cis-acting elements were relatively more abundant in number in all *BnARI* promoters (Figure 4, Tables S4 and S5). These results implied the importance of *BnARI* genes in stress adaptations.

**Figure 5:**
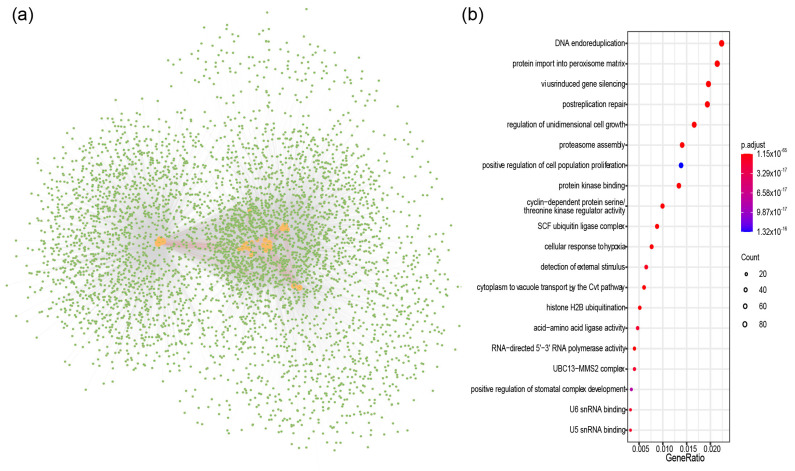
The ARI-protein interaction-network analysis in *B. napus*. (**a**) Protein–protein interaction network of BnARI proteins. The BnARI proteins are shown by orange circles, the green circles represent proteins that interacted with BnARI proteins. The red lines indicate the interaction between BnARI proteins, and the gray lines indicate the interaction between BnARI and other proteins. (**b**) KEGG pathway analysis of proteins interacted with BnARI proteins. The color bar shows the value of *p*.adjust from low (red) to high (purple).

To elucidate the functional role of *BnARIs*, we predicted the protein networks based on known protein interactions in *A. thaliana* (Figure 5, Table S6). By using 16 AtARI proteins as a query, a total of 1323 proteins were identified in the protein interactive database of *A. thaliana* that were homologous to 4714 proteins in *B. napus*. Most of the BnARI proteins interacted with each other (Figure 5a, Table S6). To further access the functional categories of these interacting proteins, Gene Ontology (GO) and Kyoto Encyclopedia of Genes and Genomes (KEGG) enrichment analyses were performed (Figure 5b, Table S6). The KEGG pathway showed that the *BnARIs* participated in the endoreduplication, post-replication repair, growth, proteasome assembly, protein transport, protein kinase activity and stress-related processes. The detailed GO analysis revealed that the aforementioned pathways were consistent biological processes terms; in the cellular component terms, mainly, the ubiquitin ligases complexes, spliseosomal complexes, autophagosome and nucleosomes were enriched (Table S6). Meanwhile, under molecular function category, protein kinase activity, cyclin-dependent protein serine/threonine kinase regulator activity, RNA polymerase activity, U5/U6 snRNA binding, Atg8 activating enzyme activity and ubiquitin ligase activity terms were enriched (Table S6).

### 2.6. Expression Patterns of BnARI Genes in Different Tissues and under Abiotic Stresses

**Figure 6:**
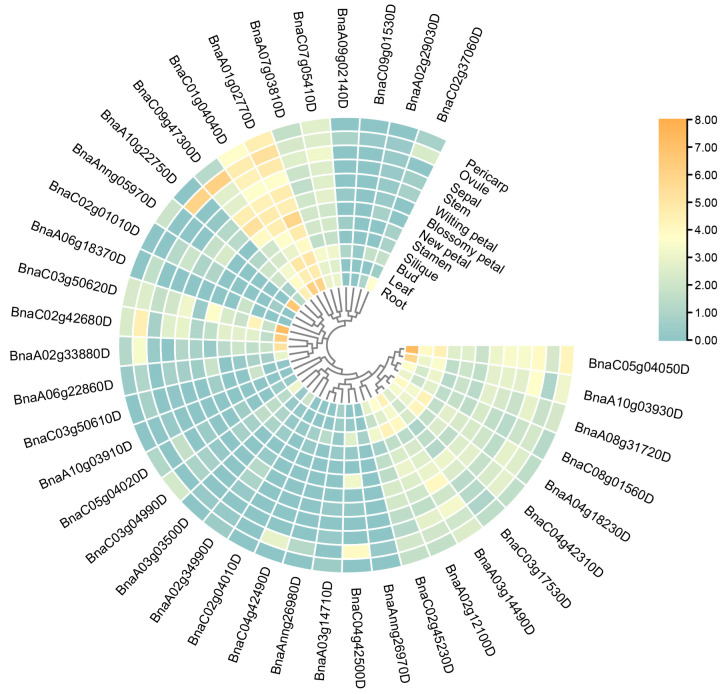
Expression profile of *ARI* genes in different tissues of *B. napus*. Heatmap was generated by taking log2 fold of FPKM values. The color bar shows relative expression from low (cadetblue) to high (orange).

We utilized the 12 tissues (root, leaf, bud, silique, stamen, new petal, blossomy petal, wilting petal, stem, sepal, ovule and pericarp) in a *B. napus* cultivar “ZS11” from our lab resources [34], to analyze the expression patterns of *BnARI* genes to predict their potential functions. We observed variable gene expressions across these tissues (Figure 6, Table S7). The results showed that the majority of genes were expressed in root and bud tissues. A total of 5 genes showed maximum expression of 70–161 FPKM in the root tissue. These genes included *BnaC01g04040D* (an ortholog of *AtARI1*) of subfamily A, *BnaC02g42680D* (an ortholog of *AtARI13*), *BnaAnng05970D* (an ortholog of *AtARI16*), *BnaC03g50620D* (an ortholog of *AtARI14*) of subfamily C and *BnaC05g04050D* (an ortholog of *AtARI5*) of subfamily B with FPKM values 72.6495, 78.5711, 152.675 and 161.35, respectively (Table S7). Two genes, *BnaA10g22750D* and *BnaAnng05970D* (an ortholog of *AtARI16*), showed predominant expression in the ovule (>60 FPKM) and one gene *BnaA01g02770D* (an ortholog of *AtARI1*) was highly expressed in the new petal tissue (60 FPKM). A total of 13 *BnARI* genes were moderately expressed among different tissues and their FPKM values ranged between 5 and 20; whereas 15 *BnARIs* were expressed below the threshold (>5) in all the tissues (Table S7). The low-expressed genes in all the tissues could be pseudogenes (Figure 6, Table S7).

There was expression variation among different tissues between the subfamilies (Figure 6, Table S7). In subfamily A, the genes were highly expressed in root > new petal > ovule. Subfamily B is the largest family of *BnARIs*, but the majority of the genes are moderately expressed in all the tissues (up to 17 FPKM). The predominant expression was observed in root > bud > pericarp (Figure 6, Table S7). All the *BnARIs* in silique tissue and orthologs of *AtARI12* in all the tissues had no FPKM greater than the threshold value; whereas, in subfamily C, all the *BnARIs* showed an FPKM below threshold in leaf, blossomy petal, stem, sepal and pericarp (Figure 6, Table S7). The predominant expression (>37) of remaining genes was observed in root > ovule > petals. We also observed the expression divergence between the duplicated genes of the same *AtARI* gene at expression level and tissue specificity; for example, in subfamily C, *AtARI16* has two duplicated pairs in *B. napus*: *BnaAnng05970D*/*BnaC02g01010D* and *BnaC09g47300D*/*BnaA10g22750D*. There was tissue-specific expression not only between each pair but also between both pairs (Figure 6, Table S7). In subfamily A, *BnaC02g37060D* (ortholog of *AtARI3*) was expressed in root tissue only, while the remaining three orthologs did not show expression in any tissue (Figure 6, Table S7). In subfamily B, the duplicated pair *BnaC04g42500D*/*BnaAnng26970D* (ortholog of *AtARI9*): the former partner was expressed in the ovule, new petal and bud (up to 13 FPKM) whereas the latter partner did not show expression in any tissue (Table S7). All these results represented clues for functional differentiation in the *ARI* gene family in *B. napus*.

**Figure 7:**
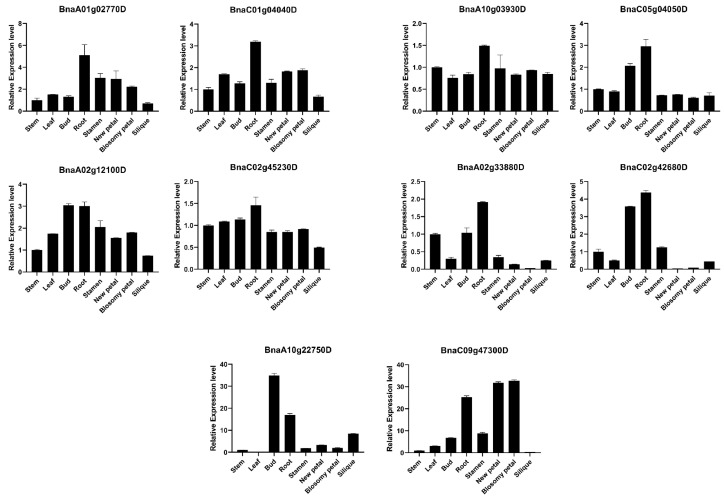
A qRT-PCR expression analysis of 10 *ARI* genes in eight different tissues of *B. napus*. The 2^−ΔΔCT^ method was used to analyze the results. The error bars indicate the standard error of the mean of three biological replicates for every tissue.

Moreover, according to predominant gene expression and the diverse expression patterns of duplicated gene pairs among various tissues, five *BnARI* duplicated genes (*BnaC01g04040D*, *BnaA01g02770D*; *BnaC05g04050D*, *BnaA10g03930D*; *BnaA02g12100D*, *BnaC02g45230D*; *BnaA02g33880D*, *BnaC02g42680D*; *BnaA10g22750D*, *BnaC09g47300D*) from the three subfamilies were selected for qRT-PCR analysis (Figure 7, Table S8). The expression pattern results verified the correctness of RNA-seq data. Expression divergence was observed in all the cases among the duplicated pairs. Most of the genes showed high expression in root tissue (Figure 7). These candidate genes must be investigated for their importance in root growth in future studies.

**Figure 8:**
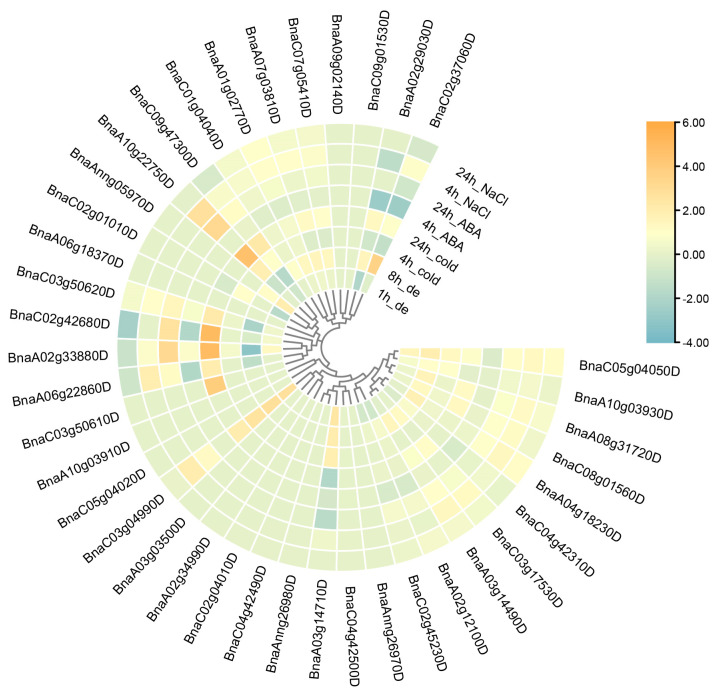
Expression profile of *ARI* genes under abiotic stress conditions in *B. napus*. The expression data is processed by comparing the control with each treated sample and calculating the log2 fold change to generate the heatmap. The color bar represents relative expression levels from low (cadetblue) to high (orange).

We also investigated the expression pattern of *BnARIs* under abiotic stresses that included dehydration, cold, ABA and salinity treatments. The transcriptome data for this analysis was retrieved from a previous study by referring to Zhang [40]. The majority of gene expressions were upregulated when the stresses were applied (Figure 8, Table S9). Thirteen genes showed a significant change in expression compared to the control (≥two folds) (Figure 8, Table S9). The expression of only one ortholog of *AtARI5*, *BnaC03g04990D*, was upregulated two folds after a 1 h and 8 h dehydration, 4 h and 24 h cold stress and 4 h saline treatment. One ortholog of *AtARI3*, *BnaC02g37060D*, displayed increased expression (3.5 fold) in 8 h dehydration stress while its expression was downregulated (−2.5 fold) under 4 h ABA treatment (Figure 8, Table S9). The orthologs of *AtARI13* displayed a significant response towards all the stress conditions. The expression of *AtARI13* orthologs (*BnaC03g50610D*, *BnaA02g33880D*, *BnaC02g42680D*, *BnaA06g22860D*) was upregulated (2–5 fold) after 24 h cold treatment (Figure S7, Table S9). The expression analysis shows the importance of these *ARI* genes during adaptation in *B. napus*.

### 2.7. Functional Importance of BnARIs Using Association Mapping Analysis in Natural Population

**Figure 9:**
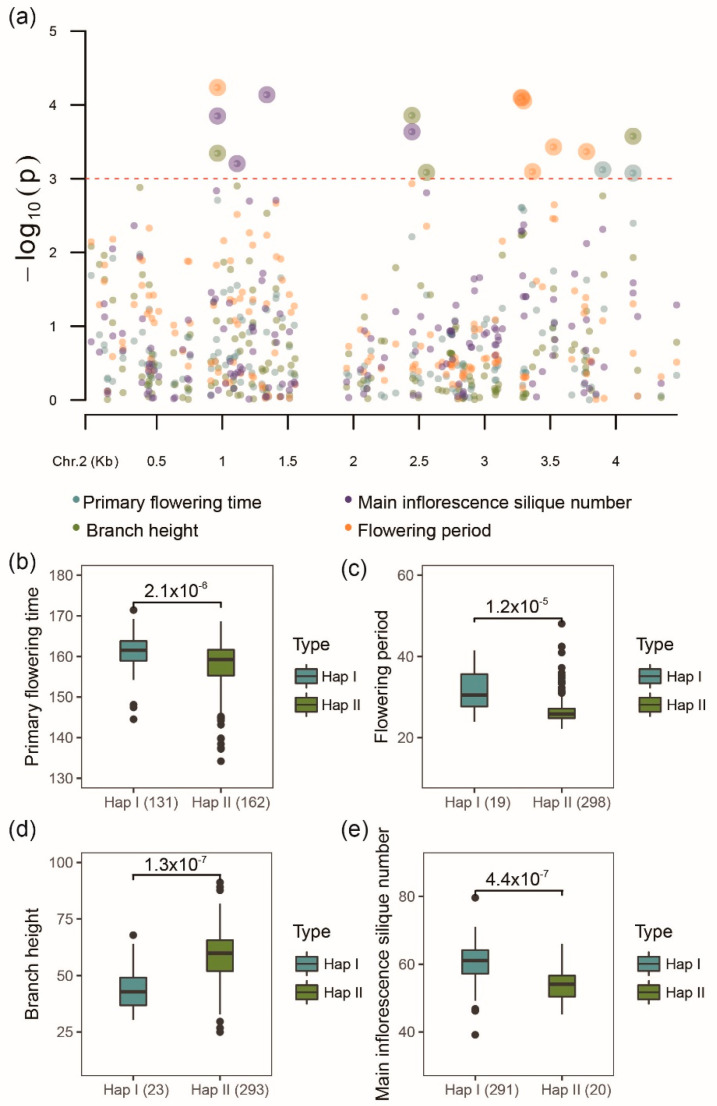
Association analysis of genetic variations in *BnaA02g12100D* in 324 worldwide collections of *B. napus* population with several agronomic traits. (**a**) Manhattan plot of *BnaA02g12100D* with primary flowering time, flowering period, branch height and main inflorescence silique number (*p* < 0.001). (**b**–**e**) The haplotype analysis of *BnaA02g12100D* genetic variation for primary flowering time, flowering period, branch height and main inflorescence silique number.

We utilized the 324 natural population accession data of our lab to find the potential effects of *BnARIs* in the agronomic traits of *B. napus*. We first analyzed their genetic variations (SNPs) and then performed association mapping analysis in natural population (Figure 9, Table S10) [41]. On average, ~40 SNPs were detected per *ARI* gene and SNP density was higher in the A subgenome (50 SNPs), whereas the SNP density was lower in the C subgenome (24 SNPs). Moreover, the average density of SNPs among the three subfamilies was in the following order: subfamily B (46) > subfamily B (42) > subfamily A (19) (Table S10). One gene, *BnaA01g02770D* from subfamily A; six genes, *BnaA10g03930D*, *BnaA04g18230D*, *BnaA03g14490D*, *BnaA02g12100D*, *BnaC02g45230D* from subfamily B; and one gene, *BnaAnng05970D* from subfamily C showed greater than 80 SNPs. There was variation in SNP number between the duplicated pairs; for example, *BnaC01g04040D*/*BnaA01g02770D* had 13/88 SNPs, *BnaA02g12100D*/*BnaA03g14490D* had 161/95 SNPs, *BnaA02g33880D*/*BnaC02g42680D* had 18/48, while *BnaA10g03930D*/*BnaC04g42500D* had 110/0 SNPs, respectively (Table S10). The SNP annotation results showed that a total of 750 SNPs were detected in the exon regions and (274) 44% of SNPs resulted in missense mutations in *BnARI* genes. These SNPs could lay the foundation of the functional importance of the *ARI* gene in *B. napus*.

In order to examine the effect of *ARI* genes on the final phenotype of the *B. napus* plant, association mapping analysis for primary flowering time (PFT), full flowering time (FFT1), final flowering time (FFT2), early flowering stage (EFS), late-flowering stage (LFS), flowering period (FP), branch number (BN), branch height (BH), plant height (PH), main inflorescence length (MIL), main inflorescence silique number (MISN), and main inflorescence silique density (MISD) were conducted. Finally, eight *BnARI* genes were identified as candidate genes for multiple agronomic traits (Table S10). Interestingly, *BnaA02g12100D* (an ortholog of *AtARI8*, *AT1G65430*) was significantly associated with nearly all traits used (*p* < 0.001) (Table S10). For flowering time, flowering period, branch height and main inflorescence silique number, the population were clearly grouped into two haplotypes, and the t-test results showed that significant differences were observed between two haplotype groups (*p* < 1 × 10^−5^) (Figure 9). Moreover, we analyzed the proteins that interacted with *BnaA02g12100D* (Table S10). The GO enrichment analysis showed that they were not only involved in ubiquitination (GO:0019005, GO:0000151, GO:0034450, GO:0034450, and so on), but also participated in multicellular organism development (GO:0007275), the negative regulation of flower development (GO:0009910), vegetative phase change (GO:0010050), the regulation of circadian rhythm (GO:0042752), meristem maintenance (GO:0010073), cellular response to auxin stimulus (GO:0071365), the maintenance of meristem identity (GO:0010074), developmental growth (GO:0048589), and the regulation of meristem development (GO:0048509) (Table S11). All these processes were related with the regulation of endogenous hormone and the development of meristem, which could finally affect flowering time and plant architecture. Overall, the results suggested that *ARI* genes could affect the agronomic traits of *B. napus*.

## 3. Discussion

The ring E3 ubiquitin ligases are widely investigated for their role in plant adaptation and development [13,14,28,42]. Since the discovery of Parkin protein that causes juvenile parkinsonism, the RBR subclass of RING-containing E3 ligases has been recognized as an essential group of proteins [18]. The plant RBR family, on the other hand, has received little attention. The RBR family has four subfamilies named as Ariadne (ARI proteins), ARA54, Plant II and Helicase in plants [16]. The Ariadne subfamily shares domain (IBR) similarity with the Parkin protein of humans. However, the functions of this subfamily are largely unidentified in plants. The Ariadne gene family is not separately explored in detail in plants except for *A. thaliana*. We performed a genome-wide investigation of Ariadne (ARI proteins) and attempted to anticipate the potential functions of this gene family in *B. napus.*

After its separation from the Arabidopsis lineage, the genus Brassica experienced genome triplication, followed by interspecific hybridization between *B. rapa* and *B. oleracea*, resulting in the allotetraploid *B. napus* [30]. As a result of these duplication events, the genome size was expanded in *B. napus* during the evolution [30] with the expectation of six genes for one *A. thaliana* gene in the *B. napus*. We identified a total of 39 *ARI* genes in *B. napus* as compared with 16 *AtARI* genes (Table 1). The expansion of this gene family was only about 2.5 fold more than the ancestor *A. thaliana*, representing gene loss [43]. We did not obtain any ortholog against two *AtARI* genes (*AtARI6* (*At1g63760*), *AtARI4* (*At3g27720*) which were considered as pseudogenes [9]). Many studies showed that WGD or segmental duplication play a critical role during the expansion of gene families [44,45,46]. We obtained similar results in *BnARI* genes: all the genes were produced from the WGD or segmental duplication except four genes *BnaAnng26970D*, *BnaAnng26980D*, *BnaC04g42500D*, *BnaA03g14710D* and *BnaC03g50620D*, which were produced as a result of tandem duplication events (Table 1). The ka/Ks ratio was less than one for all duplicated pairs, suggesting purifying selection during the evolution. There was variation in Ka/Ks for the duplicated pairs, suggesting that they had evolved at different rates of evolution. The average divergence time for all the duplicated pairs was about 4 MYA, suggesting recent duplications in the *BnARI* gene family, which was also consistent with the evolutionary process of *B. napus*.

The evolution and differentiation of genes was deduced by comparing protein homologies, gene structure and motif combinations. We used this way to classify the *BnARI* gene family. Using Arabidopsis ARI proteins as a reference, these ARI proteins were clustered into three subfamilies (A, B and C). This classification was further confirmed by gene structure and motif analysis within the *BnARI* gene-family members. The number of exon and introns within subfamily C was conserved but exon and intron number was not conserved within subfamily A and B. Then, the investigation of motifs revealed that the coding sequence was highly conserved within the subfamilies, suggesting that the coding sequences were conserved in subfamily A, B and C.

Based on Cello server prediction, almost all the proteins were localized to a nuclear region, and these results were supported by a previous study [9], except for *BnaA02g33880D* and *BnaC02g42680D*, which were localized to an extracellular region. The possible explanation for this observation was that only these two proteins did not contain motif 7, which encoded for the Ring finger (Figure 3, Table S3). However, further functional studies are required to decode the mechanism.

To determine the possible function of *BnARI* genes, we investigated the cis-elements in their promoters that could influence their expression pattern [47] (Figure 4, Tables S4 and S5). In the RNA-seq data of twelve tissues, which included the root, leaf, bud, silique, stamen, new petal, blossomy petal, wilting petal, stem, sepal, ovule and pericarp of blooming of *B. napus* [34], interestingly, the highest gene expression was observed in the root tissue (Figure 6, Table S7). Mostly, genes were expressed in the root and bud tissue and the fewest genes were expressed in the silique tissue. We observed differential expression patterns of all the genes among different tissues, as discussed in the Results section (Figure 6, Table S7). Expressional divergence was observed between the duplicated gene pairs as well, suggesting a clue for sub/neo functionalization and pseudogenization, like in other polyploid crops [48]. Plants face several kinds of external stimuli including light, drought, high temperature, influencing their growth and development. Therefore, they evolve many strategies to cope with these challenges [49]. In the promoters of *BnARIs*, we detected various type of stress-related cis-elements (Figure 8, Table S4) that could predict their importance in abiotic stress. In the RNA-seq data for four stress treatments (dehydration, cold, ABA and salinity), subfamily C, especially all the orthologs (*BnaC03g50610D*, *BnaA02g33880D*, *BnaC02g42680D*, *BnaA06g22860D*) of *AT5G63750*, (*AtARI13*) showed significant responses towards the stresses applied (Figure 8, Table S9). The previous studies also showed the importance of *ARI* genes against abiotic stress in plants [26,27,28]. The gene ontology (GO) and KEGG pathway analyses predicted the involvement of *BnARIs* in endoreduplication (polyploidy), DNA damage, kinase activity, proteasome assembly, ubiquitination and stress mechanisms (Figure 5, Table S6). E3 ligases are also known to be involved in the aforementioned processes [7].

We also investigated genetic variations (SNPs) for *BnARIs* in the natural *B. napus* population [41] (Table S10). The greater number of SNPs in the genomic regions suggested that a lot of variations occurred in *BnARIs* during the evolution. The SNP density was much higher in the A subgenome than the C subgenome (Table S10) and these results were consistent with the *SR* and *GATA* gene families in *B. napus* [45,50]. In this study, a total of 271 missense mutations could help in the functional differentiation of these *BnARI* genes. We further used these genetic variations and performed association analysis with different agronomic traits to predict the importance of *ARI* genes in *B. napus* (Figure 9). Among all the genes, *BnaA02g12100D* (ortholog of *ARI8*, *AT1G65430*) was significantly associated with nearly all the traits used (threshold 3) (*p* < 0.001) (Figure 9, Table S10). This gene has a high and more diverse expression pattern than the other duplicated partner (*BnaC02g45230D*) in our transcriptome studied data (Figure 6, Table S7). In addition, its expression variation was observed in all the stresses as compared with the control. Based on the GO enrichment analysis for its interacted protein, it could be involved in diverse biological processes including ubiquitination, phosphorylation of proteins, meristem development and response to hormones (Table S11), which eventually influence the phenotype and adaptation of plants. According to a previous study, when expression analysis was carried out among *RBR* genes in 79 developmental stages of *A. thaliana*, *ARI8* (ortholog of *BnaA02g12100D)* was the predominantly expressed gene and its expression was specifically detected to be high in the mature pollen stage [16]. Among the 103 tissues of several developmental stages in *B. napus*, the expression of *BnaA02g12100D* in mature anther was specifically high [35]. Expression specificity in the male gametophyte could influence plant fitness and phenotype by causing the proteins to become ubiquitinated [51,52]. Among all the AtARI proteins, ubiquitin activity was only proven in *AtARI8* [6]. Ubiquitination is one of the critical mechanism that controlled the photoperiodic pathway in flowering regulation [14]. Likewise, the E3 ligases were involved in the regulation of some important flowering time genes, for example, constans, constitutive photomorphogenic 1, and target of early activation tagged 2 [53]. The knockout mutants of their orthologous genes in animals were lethal [24]. Nevertheless, the knockout studies for *ARI* genes are not available in any plants and the role of *ARI* genes in development and stress tolerance should be further explored. Therefore, these findings can provide a better clue to understand the significance of *BnARIs* in phenotypic variation and adaptation.

## 4. Materials and Methods

### 4.1. Identification of ARI Gene Family in B. napus

To identify the *BnARI genes*, we performed the BLASTP search in *B. napus* at BnaOmics database (Available online: https://bnaomics.ocri-genomics.net, accessed on 6 February 2022), 16 AtARI protein sequences were used as queries with e-value 1× 10^−5^. The NCBI Conserved Domain Database (Available online: https://www.ncbi.nlm.nih.gov/cdd, accessed on 6 February 2022) [54] and Pfam database (Available online: http://pfam.xfam.org/, accessed on 6 February 2022) [55] were used for verification of candidate genes that contained IBR and Ariadne domain. The redundant genes were removed manually.

The genome data files of “*Darmor*-bzh” were used to collect sequence information for all *BnARIs*, including ID, CDS, proteins, chromosomal position at BnaOmics database (Available online: https://bnaomics.ocri-genomics.net, accessed on 6 February 2022) [30]. The ExPasy tool (Available online: http://www.expasy.org/, accessed on 6 February 2022) was used to compute the peptide length (aa), molecular weight (MW), and isoelectric point (PI) of each BnARI protein. CELLO v2.5 (Available online: http://cello.life.nctu.edu.tw/, accessed on 6 February 2022) [56] was used to predict the subcellular localization of BnARI proteins.

### 4.2. Phylogenetic Analysis of BnARI Family

Multiple sequence alignments of ARI proteins from *A. thaliana* and *B. napus* were performed by using the ClustalW v2 [57] programme to acquire insights into the phylogenetic relationships between ARI family members. The neighbor-joining (NJ) technique was used with 1000 bootstrap replications in the MEGA v11 [58] to create the phylogenetic tree. Furthermore, iTOL v6.5.2 (Available online: https://itol.embl.de/, accessed on 12 February 2022) was used to visualize the tree. According to homology, *ARI* genes were further subdivided into subfamilies.

### 4.3. Chromosomal Distribution, Duplication Status, Ka/Ks Ratio

The physical positions of the *BnARI* genes on chromosomes were identified using the *B. napus* genome annotation file in TBtools programme, v1.098 [59]. BLASTP, with e-value of 1 × 10^−10^ and MCScanX [60], was used to evaluate the duplication patterns, such as segmental and tandem duplications. The TBtools programme v1.098 [59] was used to visualize the chromosomal location and duplicated *BnARI* genes.

The ratios of synonymous substitution rate (ks) and non-synonymous substitution rate (ka) of homologous *BnARI* gene pairs were calculated using TBtools programme (v1.098) [59]. T = Ks/2R, where R is 1.5 × 10^−8^ synonymous substitutions per site per year, was used to calculate divergence [61]. Ka/Ks ratio less than one indicated purifying selection, whereas Ka/Ks ratio greater than one indicated positive selection.

### 4.4. Identification of Conserved Motifs and Gene Structure in BnARI Gene Family

The conserved motifs in the BnARI proteins were analyzed using Multiple Expectation Maximization for Motif Elicitation (MEME 5.4.1) [37]. The parameters were organized into 10 motifs with widths between 6–50 amino acids and the remaining options were left at default settings. The Pfam database (Available online: http://pfam.xfam.org/search, accessed on 15 February 2022) was used to annotate the discovered motifs. The gene-structure information was obtained from *B. napus* genome files. The gene and motif structures were visualized using the TBtools programme, v1.098 [59].

### 4.5. Identification of Cis-Acting Regulatory Elements and Protein–Protein Interaction in BnARI Gene Family

To find the cis-acting regulatory elements in the *BnARI* genes promoters, 2 kb upstream each gene was extracted and examined through PlantCARE database (Available online: http://bioinformatics.psb.ugent.be/webtools/plantcare/html/, accessed on 15 February 2022) [38]. The number of cis-acting elements were converted to log2 transformation and were visualized using TBtools programme, v1.098 [59].

To determine the protein–protein interactions (PPIs) of BnARIs, first PPIs of the AtARI proteins were downloaded from STRING database (Available online: https://www.string-db.org/, accessed on 15 February 2022) [39], the BnARI-interacting proteins were predicted based on the homologs in *A. thaliana*, and the network was visualized using Cytoscape [62]. Using the clusterProfiler in R [63], the genes that interacted with the BnARI proteins were selected for gene ontology and KEGG enrichment analysis.

### 4.6. Expression Analysis of BnARI Genes in Different Tissues and under Abiotic Stress

We used transcriptome data from twelve tissues of “ZS 11” (root, leaf, bud, silique, stamen, new petal, blossomy petal, wilting petal, stem, sepal, ovule and pericarp) [34] and four treatments (dehydration, cold, ABA and salinity) to detect the expression of *BnARI* genes [40]. The FPKM values were transformed to log2 folds, and TBtools was used to produce heatmaps of all data.

### 4.7. RNA Extraction and Reverse Transcription-Quantitative PCR (qRT PCR)

The *B. napus* v. “ZS 11” was grown in Oil Crop Research Institute (OCRI) Wuhan fields. Three samples for each tissue (leaf, stem, root, petal, sepal, carpel, and stamen) were collected at the blooming stage. The 21 samples were immediately put in liquid nitrogen before storing at −80 °C. The Invitrogen TRIZOL Reagent (Thermofisher, Waltham, MA, USA) was used to isolate total RNA. A TaKaRa reverse transcription kit (Prime ScriptTMRT reagent Kit, TaKaRa, Beijing, China) was used to make first-strand complementary DNA (cDNA). Furthermore, qRT-PCR primers for potential *BnARI* genes were constructed to test the expression pattern (Table S8). The qRT-PCR was performed using Bio Supermix (Bio-rad, Hercules, CA, USA) according to the manufacturer’s instructions, with reaction steps as follows: 95 °C for 3 min; 40 cycles of 95 °C for 15 s; 56 °C for 15 s, followed by 65 °C for 5 s and 95 °C for 5 s in three biological replicates. Ten genes of 5 duplicated pairs were analyzed for expression by qRT-PCR. The *B. napus*—β actin gene (*AF111812*) was used as internal reference. The 2^−∆∆Ct^ method [64] was used to compute the relative expression.

### 4.8. Functional Significance of BnARIs by Using Association Mapping in Natural Population

To determine the functional significance of *BnARIs* at the population level, we investigated the natural genetic variations (SNPs) in them using 324 accessions, which were collected from around the world [41]. The SNPs were obtained and annotated through SnpEff programme v4.11 [65]. Primary flowering time (PFT), full flowering time (FFT1), final flowering time (FFT2), early flowering stage (EFS), late-flowering stage (LFS), flowering period (FP), branch number (BN), branch height (BH), plant height (PH), main inflorescence length (MIL), main inflorescence silique number (MISN), and main inflorescence silique density (MISD) were selected as studied traits [41]. EMMAX [66] was used to perform the association mapping analysis between genetic variations and agronomic traits. CMplot and ggplot2 in R (Available online: https://mirrors.tuna.tsinghua.edu.cn/CRAN/, accessed on 15 February 2022) were used to draw the Manhattan plot and the boxplot.

### 4.9. Statistical Analysis

The statistical analysis was performed by R (v3.6) and means of two continuous normally distributed variables were compared by independent samples Student’s T-test. A value of *p* < 0.05 was considered significant.

## 5. Conclusions

In this study, the detailed investigation and characterization of Ariadne genes was carried out. All 39 *ARI* genes were classified into three subfamilies. Gene architectures and motifs were comparable across genes from the same subfamily. The presence of cis-acting regulatory elements in the *ARI* genes promoters, as well as their expression patterns in diverse tissues and under various environmental conditions, and the protein interaction analysis demonstrated that they could play an important role in development and stress tolerance. Furthermore, genetic variations in *BnARI* genes provided diverse potential influences on agronomic traits. In summary, this study supplied useful information on *BnARI* genes, and it will aid further functional research and genetic improvement for breeding in *B. napus*.

## Figures and Tables

**Table 1:** Characteristics of the *ARI* genes in *B. napus* (pI, isoelectric point; MW, molecular weight).

ID	Classification	pI	MW (kDa)	Amino acids	Chromosome	Start	End	Duplication Type	Exon Number	Subcellular Localization
BnaC01g04040D	subfamily A	5.44	68.30577	598	C01	2107232	2110597	WGD or Segmental	6	Nuclear
BnaA01g02770D		5.49	67.70018	592	A01	1363570	1366772	WGD or Segmental	6	Nuclear
BnaA07g03810D		5.5	66.84028	583	A07	3464224	3466892	WGD or Segmental	7	Nuclear
BnaC07g05410D		5.39	67.01159	582	C07	8591383	8594140	WGD or Segmental	7	Nuclear
BnaA09g02140D		5.38	61.03602	525	A09	1051875	1053449	WGD or Segmental	1	Nuclear
BnaA02g29030D		5.28	55.36126	481	A02	21265416	21266987	WGD or Segmental	3	Nuclear
BnaC09g01530D		5.45	56.70796	485	C09	836916	838508	WGD or Segmental	3	Nuclear
BnaC02g37060D		5.07	55.77174	485	C02	40014335	40015918	WGD or Segmental	3	Nuclear
BnaC02g04010D	subfamily B	5.53	58.47465	510	C02	2040175	2041821	WGD or Segmental	2	Nuclear
BnaC05g04050D		5.12	63.11043	556	C05	1976038	1981190	WGD or Segmental	15	Nuclear
BnaA02g34990D		5.83	62.01988	541	A02_random	24816	26477	WGD or Segmental	2	Nuclear
BnaA10g03930D		5.11	63.19455	559	A10	2088735	2093862	WGD or Segmental	15	Nuclear
BnaA03g03500D		5	56.69741	496	A03	1688116	1689720	WGD or Segmental	2	Nuclear
BnaC03g04990D		5.02	59.7861	526	C03	2416072	2417649	WGD or Segmental	1	Nuclear
BnaA04g18230D		4.99	63.39872	556	A04	14658509	14662929	WGD or Segmental	15	Nuclear
BnaC03g17530D		4.9	63.69074	558	C03	8960359	8965066	WGD or Segmental	15	Nuclear
BnaC04g42310D		4.92	63.61489	558	C04	42886627	42891037	WGD or Segmental	15	Nuclear
BnaA08g31720D		5.08	62.69513	554	A08_random	2100605	2104866	WGD or Segmental	15	Nuclear
BnaA03g14490D		4.85	63.39747	555	A03	6669544	6674154	WGD or Segmental	15	Nuclear
BnaC08g01560D		5.03	62.76486	555	C08	1211774	1215865	WGD or Segmental	15	Nuclear
BnaA02g12100D		5.06	62.48365	548	A02	6326435	6331272	WGD or Segmental	16	Nuclear
BnaC02g45230D		5.05	64.28373	565	C02_random	956624	961488	WGD or Segmental	15	Nuclear
BnaAnng26970D		4.96	57.65184	499	Ann_random	30922371	30924172	Tandem	2	Nuclear
BnaAnng26980D		6.48	51.01917	440	Ann_random	30926298	30927929	Tandem	2	Nuclear
BnaC04g42500D		5.09	63.14806	548	C04	43066664	43068633	Tandem	2	Nuclear
BnaA03g14710D		5.83	60.14362	522	A03	6761186	6762872	WGD or Segmental	2	Nuclear
BnaC04g42490D		5.39	66.50624	574	C04	43060145	43062382	WGD or Segmental	3	Nuclear
BnaC05g04020D		5.18	61.60797	529	C05	1967024	1969723	WGD or Segmental	14	Nuclear
BnaA10g03910D		5.28	60.45582	522	A10	2073034	2075988	WGD or Segmental	15	Nuclear
BnaC03g50610D	subfamily C	4.87	75.1735	657	C03	35066895	35072312	WGD or Segmental	3	Nuclear
BnaA02g33880D		5.15	51.08088	446	A02	24242394	24244388	WGD or Segmental	2	Extracellular
BnaC02g42680D		5.31	51.23706	447	C02	45189629	45191512	WGD or Segmental	2	Extracellular
BnaA06g22860D		4.7	61.10942	532	A06	16009537	16013955	WGD or Segmental	3	Nuclear
BnaC03g50620D		4.82	42.64821	369	C03	35073684	35074888	Tandem	2	Nuclear
BnaC02g01010D		5.61	54.80394	483	C02	436156	437604	WGD or Segmental	1	Nuclear
BnaA06g18370D		4.99	50.6674	447	A06	10552571	10553998	WGD or Segmental	2	Nuclear
BnaA10g22750D		5.06	74.29611	672	A10	15235470	15237795	WGD or Segmental	3	Nuclear
BnaAnng05970D		5.38	54.8181	478	Ann_random	6115791	6117224	WGD or Segmental	1	Nuclear
BnaC09g47300D		6.05	73.81982	665	C09	46717181	46719562	WGD or Segmental	4	Nuclear

## Data Availability

The corresponding data are shown in Supplementary Materials.

## Supplementary Materials

The following are available online at https://www.mdpi.com/article/10.3390/ijms23116265/s1.
